# Green Synthesis of Antibacterial Nanocomposite of Silver Nanoparticle-Doped Hydroxyapatite Utilizing *Curcuma longa* Leaf Extract and Land Snail (*Achatina fulica*) Shell Waste

**DOI:** 10.3390/jfb13020084

**Published:** 2022-06-20

**Authors:** Is Fatimah, Habibi Hidayat, Gani Purwiandono, Khoirunisa Khoirunisa, Hasna Azizah Zahra, Rahmania Audita, Suresh Sagadevan

**Affiliations:** 1 Department of Chemistry, Faculty of Mathematics and Natural Sciences, Universitas Islam Indonesia, Kampus Terpadu UII, Jl. Kaliurang Km 14, Sleman, Yogyakarta 55584, Indonesia; habibihidayat13@uii.ac.id (H.H.); gani.purwiandono@uii.ac.id (G.P.); 18612122@students.uii.ac.id (K.K.); hasna.zahra@students.uii.ac.id (H.A.Z.); rahmania.audita@students.uii.ac.id (R.A.); 2 Nanotechnology & Catalysis Research Centre, University of Malaya, Kuala Lumpur 50603, Malaysia; drsureshnano@gmail.com

**Keywords:** hydroxyapatite, silver nanoparticles, antibacterial nanocomposite

## Abstract

Preparation of green synthesized silver nanoparticle (AgNPs)-doped hydroxyapatite (Ag/HA) utilizing *Curcuma longa* leaf extract and land snail (*Achatina fulica*) shell waste was performed. Physicochemical characteristics and antibacterial activity of Ag/HA composite as a function of Ag content was studied. Instrumental analysis such as XRD, SEM-EDX, TEM, and XPS were employed to characterize the nanocomposites. The physicochemical study revealed the maintained porous structure of HA after Ag immobilization, and from TEM analyses, it was found that the distributed spherical particles are associated with the dispersed Ag and have a particle diameter of around 5–25 nm. Antibacterial activity of the nanocomposite was evaluated against *Escherichia coli*, *Staphylococcus aureus*, *Kliebsiella* *pneumonia*, and *Streptococcus pyogenes*. The results showed that the varied Ag content (1.0; 1.6; and 2.4% wt) influenced the nanoparticle distribution in the nanocomposite and enhanced the antibacterial feature.

## 1. Introduction

Silver nanoparticles (AgNPs) have received intensive attention due to their vast applications in the biomedical field, i.e., as antibacterial, antifungal, and anticancer agents [1]. The uses of AgNPs-based materials for such medical uses as in artificial bone and teeth, ceramic coating, and other sanitizing installations, are already popular in commercial use. Within these schemes, the addition of AgNPs to composite materials such as chitosan, graphene, and hydroxyapatite (HA) demonstrated some beneficiary advantages for certain purposes [2,3]. Among possible combinations, substituted HA with AgNPs was widely developed to extend its biomimetic applications with antioxidative, antibacterial, anti-fungi, and anti-virus features. The antibacterial and antioxidative activity of the composites are useful for various medical applications, and thus the explorations were attempted from many perspectives [4,5]. Several research studied the use of Ag-doped HA for various medical devices; for example, they are used as a bone implant with an improved osteoconductivity [6], and as bioceramic layers with antifungal and antibacterial properties [7,8]. In biosensing application, Ag-doped HA was reported to be potential applied for trace-level formaldehyde detection [9].

The interests for Ag-doped HA synthesis are related to the composition of dopant in the nanocomposite–due to its toxicity at a certain level–and to the effectiveness of the synthesis procedure, cost, and materials characteristics [10]. One of the critical developments is to minimize the use of hazardous chemicals by the green synthesis approach, either in the synthesis of AgNPs or synthesis of HA. The synthesis via bioreduction of Ag^+^ precursor by secondary metabolites from many valuable plants is a well-known and emerging research [11,12,13]. The uses of secondary metabolites from medicinal plants extracts as bio-reductors are the popular pathway for this; more than 73,000 publications have been reported to synthesize AgNPs with certain forms with specific bioactivity performance [14,15]. In addition, HA synthesis utilizing CaO derived from waste minerals aims to achieve a similar goal. From several publications, biogenic calcium (CaO) derived from *Molluscan* shell waste–including land snail shell–for HA synthesis is a potential low-cost option for sustainable material development [16,17]. Based on these ideas, combining green synthesized AgNPs and HA to get an antibacterial and antioxidative active composite is an exciting perspective that needs further explorations [14].

Based on previous investigations, it was revealed that extract from *Curcuma longa* tuber and leaf powder is inspiring for AgNPs synthesis due to its bioactivity and bio-reductivity [18,19]. The huge potency of *C. longa* in traditional medicine in some countries, including in Indonesia, will become a distinctive innovation for further important research schemes in nanocomposites for biomimetic and other medical purposes. Based on these factors, this research aims to study the physicochemical characteristics and antibacterial features of Ag-doped HA (from herein called Ag/HA) utilizing *C. longa* leaf extract, (CLE)-mediated synthesized AgNPs, and land snail shell waste as biogenic calcium for HA. As the renewable resources of bioreductor and biogenic calcium utilized in the synthesis, it can be denoted as the green synthesis method. The focus of the studies were on the effect of the AgNPs content on the antibacterial activity of Ag/HA. The antibacterial activity of AgNPs was evaluated on *Escherichia coli* (ATCC 11303), *Staphylococcus aureus* (ATCC 25923), *Kliebsiella pneumonia* (ATCC 11828), and *Streptococcus pyogenes* (ATCC 87110) due to the infectious effect and impact of these bacteria on humans and the environment. All bacteria were obtained from Microbiology Laboratory, Universitas Islam Indonesia. 

## 2. Materials and Methods

### 2.1. Materials

Land snail (*Achatina fulica*) shell waste was obtained from a cultivation area in Sleman District, Yogyakarta Province, Indonesia. Fresh *C. longa* leaves were collected from the Universitas Islam Indonesia Park. The identification and authentication of plant taxonomy were performed at the Biology Department, Universitas Gadjah Mada, Yogyakarta, Indonesia. About 10 g of washed leaves were crushed and soaked in aquadest for 4 h and filtered to obtain *C. longa* leaf extract (called CLE). Biogenic calcium (CaO) was derived by calcining crushed land snail shells at 1050 °C [20,21]; previous investigation revealed the single phase of CaO obtained by this treatment. Chemicals consisting of AgNO_3_ (>99%) and (NH_4_)_2_HPO_4_ (99%) were purchased from Merck (Darmstadt, Germany).

### 2.2. Synthesis of AgNPs

The AgNPs was synthesized by mixing 9 mL of AgNO_3_ 10^−3^ M with 1 mL of CLE, followed by microwave heating for 15 min. The reduction mechanism was monitored by UV-Visible spectrophotometry and particle size distribution identifications. Physicochemical characterization of synthesized AgNPs was conducted by UV-Visible spectrophotometer analysis based on the identification of surface plasmon resonance of AgNPs, with additional TEM analysis to determine the nanoparticle sizes and distribution.

### 2.3. Synthesis of Ag/HA

The Ag/HA samples were prepared utilizing AgNPs solution and CaO derived from land snail shells by slow mixing and setting with the atomic ratio of Ag/[Ag+ Ca] at 1.0; 1.6; 2.4%. Furthermore, the Ca with AgNPs solution was added with (NH_4_)_2_HPO_4_ to obtain [Ca+ Ag]/P as 1.67 in a total 250 mL solution. The range of Ag percentage was determined based on previous investigations considering the range of Ag content significantly influences the antibacterial activity of the Ag/HA [22,23,24]. The solution was stirred for 2 h before it was hydrothermally treated in an autoclave at 150 °C overnight. The obtained slurry was then dried in an electric oven at 80 °C before sintering at 750 °C for 1 h. The obtained samples were encoded as Ag1.0/HA, Ag1.6/HA, and Ag2.4/HA for the varied Ag concentrations. There was no residue left from these syntheses.

### 2.4. Physicochemical Characterization of Ag/HA

The physicochemical characterization of the nanocomposites was investigated by using X-ray diffraction (XRD)(Bruker AXS D8, Singapore), Scanning Electron Microscope-energy dispersive X-ray (SEM-EDX) (Phenom-X, Singapore), transmission electron microscopy (TEM), and X-ray photoelectron spectroscopy (XPS). For XRD analysis, a Shimadzu X6000 diffractometer (Singapore) operated using Cu Kα radiation at λ = 0.154 nm was employed, and the peaks were recorded at the range of 5–80°. For surface morphology and elemental analyses, SEM-EDX was operated on Au-coated samples. The analysis was conducted by a magnification of 10,000×. Transmission electron microscopy (TEM) identification was performed on a JEOL-JEM-2100 (Tokyo, Japan) operated at 20 kV, and a ULVAC instrument (Quantera SXM, Japan) was used for XPS analysis.

### 2.5. Antibacterial Activity Test of Ag/HA

A nutrient medium for the bacterial culture was utilized for bacterial growth and prepared by suspending nutrient agar in distilled water and autoclaved before use. About 5 μL of Ag/HA suspension solution was loaded onto a 6 mm filter disc, and bacterial culture was evenly spread throughout the petri plate. The evaluation of the antibacterial activity was performed based on the inhibition zone of each sample after 24 h incubation. The minimum inhibitory concentration (MIC) of each sample was performed by the dilution method. The inhibition zone at varied Ag/HA concentration was measured, and the MIC was determined by the smallest concentration giving significant antibacterial activity.

## 3. Results

### 3.1. Synthesis of AgNPs

**Figure 1:**
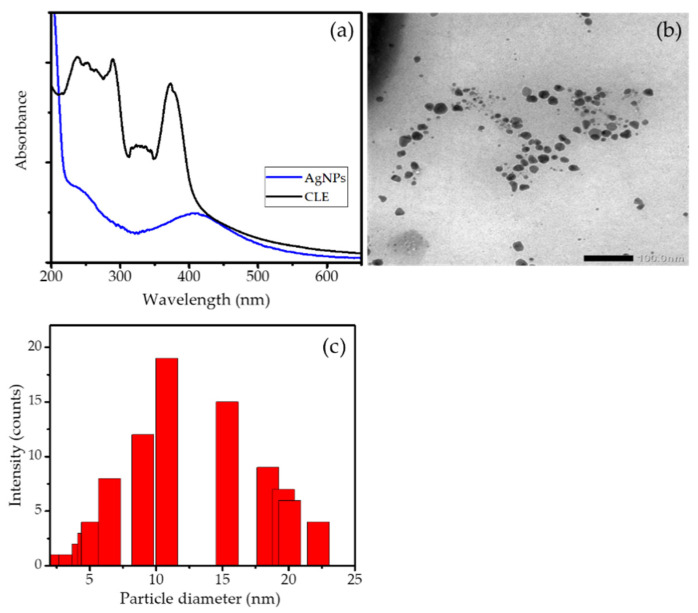
(**a**). UV-Visible spectra of CLE and AgNPs (**b**). TEM image of synthesized AgNPs (**c**) Particle size distribution of AgNPs.

The synthesis of AgNPs is the first step of the Ag/HA preparation. The principle of AgNPs synthesis is the bioreduction of Ag^+^ from AgNO_3_ into Ag^0^ by the secondary metabolite from CLE. Figure 1a presents the UV-Visible spectrum of CLE and the formed AgNPs as the proof of bioreduction. The CLE spectrum exhibits some peaks at the range of 200–400 nm as the indication of phenolic compounds from such secondary metabolites contained in CLE. As the result of Ag^+^ reduction, these peaks associated with CLE compounds disappear, with instead a single peak at 410 nm that is characteristics of the surface plasmon resonance spectrum of AgNPs [25]. The similar spectrum was also reported in the AgNPs synthesis by using CLE [18,26]. The formation of nanoparticles was also confirmed by the TEM image of the formed particles and the particle size distribution presented in Figure 1b,c, respectively. In addition, TEM analysis was performed for the synthesized AgNPs. The TEM image in Figure 1b represents a heterogeneous form of the nanoparticles, with the particle sizes ranging from 2–20 nm. The particle size distribution depicted in Figure 1c implies a particle size mean of around 12 nm.

### 3.2. Characterization of Ag/HA

**Figure 2:**
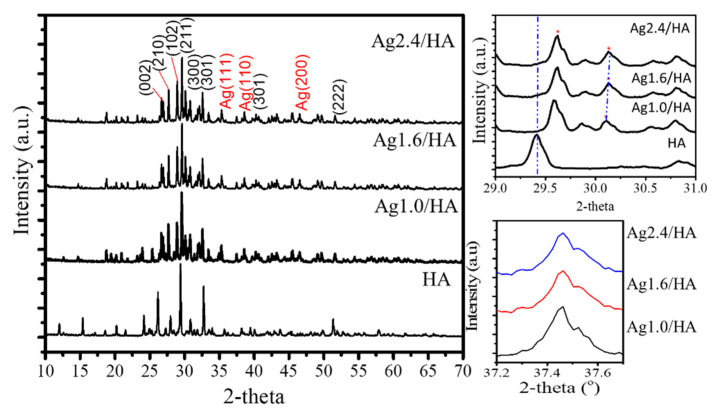
XRD pattern of nanocomposites.

The XRD patterns of Ag/HA samples are shown in Figure 2. The presence of the crystalline structure of HA is identified by the planes of (002), (210), (211), (112), (300), (310), (222), and (213); refer to the JCPDS card no. 09–432 [27,28]. Reflections are still maintained in Ag/HA samples other than the existence of Ag reflections presented at around 37.5, 39.0, and 47.4°, which are associated with planes (111), (110), and (200) (refer to JCPDS No: 84–7103), respectively. From the compared (211) plane of HA, a shifted reflection to a higher angle due to the Ag doping is found, which indicates the replacement of Ca ions with Ag ions [29,30]. Moreover, the shift is not significant due to the increasing Ag content. In addition, using the Scherer calculation for the (111) plane of Ag, the crystallite size of Ag is not appreciably different, valued at about 87.7–88.9 nm.

**Figure 3:**
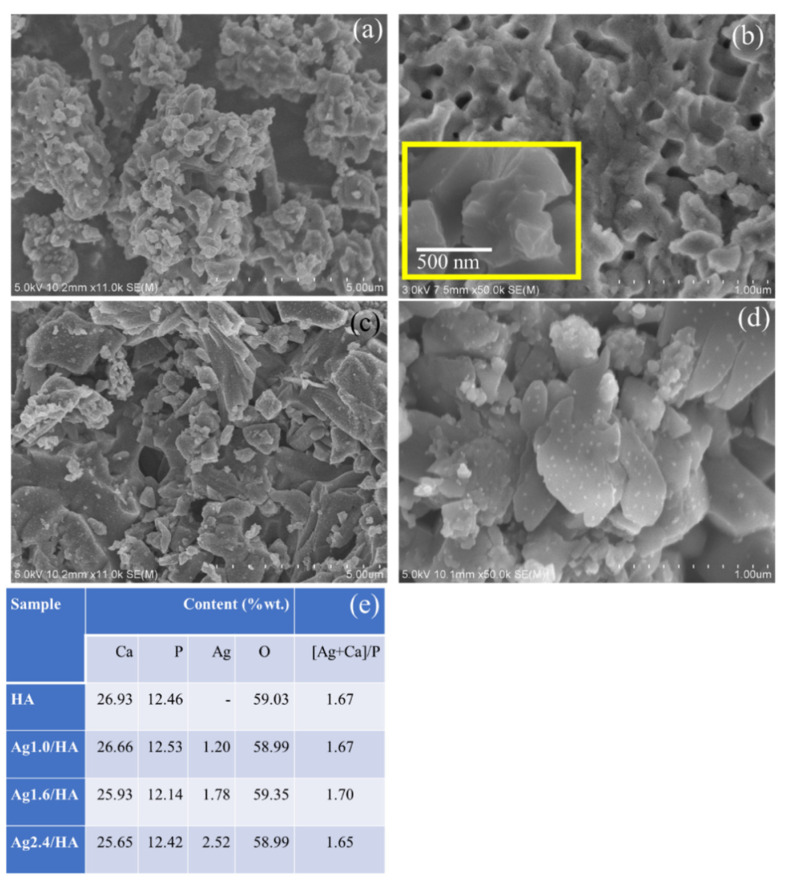
(**a**,**b**) SEM images of HA (**c**,**d**) SEM images of Ag/HA, respectively (**e**). EDX results.

**Figure 4:**
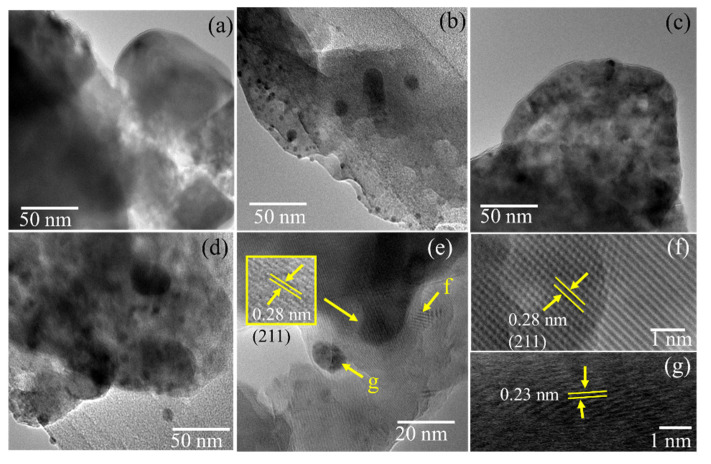
(**a**–**d**) TEM images of HA, Ag1.0/HA, Ag1.6/HA, and Ag2.4/HA (**d**–**g**) HRTEM of Ag/HA.

Figure 3 shows the surface morphology of HA and Ag/HA samples. A distributed AgNPs identified by white dots is exhibited by Ag/HA samples, which was also confirmed by the elemental analysis from XRD analyses presented in Figure 3e. It was also found that the Ag contents in the nanocomposite are relatively matched with what was set up, and so are the Ca/P ratios at around 1.65–1.70. The more detailed morphology revealed the porous structure of HA, which is maintained by the modification. The more detailed analysis of the doped Ag was performed by TEM analysis, with the images presented in Figure 4.

The high-resolution TEM images in Figure 4f highlighted well-resolved lattice fringes, which are observed at the distances of 0.35 and 0.28 nm that are associated with the d_002_ and d_211_ of HA structure, respectively. In addition, the distributed spherical particles are associated with the dispersed Ag have a particle diameter of around 5–25 nm. The lattice fringes of the spot presented in Figure 4g represent the distance of 0.23 nm, which is associated with the (111) plane of Ag [31,32]. From the different Ag content, the higher particle diameters and the density appeared at higher Ag concentrations. The relatively controlled Ag substitution was identified at lower AgNPs amount in the preparation step.

**Figure 5:**
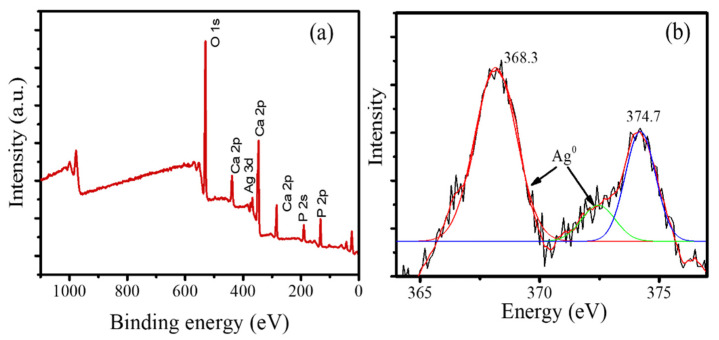
(**a**). Survey scan spectrum of Ag/HA (**b**). Spectrum of Ag 3d.

However, XPS analysis of the samples exhibits a single species of Ag^0^ in the structure, as confirmed by the Ag 3d spectrum presented in Figure 5. The survey scan in Figure 5a reveals some peaks associated with the existence of Ca, P, and O atoms as the main components of HA [33]. The calculation of the intensity ratio of the Ca/P peaks shows 1.667, which is within the ratio obtained by EDX analysis and meets the theoretical ratio in HA (1.67). In addition, the Ag 3d spectrum shows double peaks at 368.3 and 374.7 eV associated with 3d_5/2_ and 3d_3/2_, respectively. These peaks correspond to unperturbed metallic silver, as expected for Ag in nanoparticle form [34].

### 3.3. Antibacterial Activity

**Figure 6:**
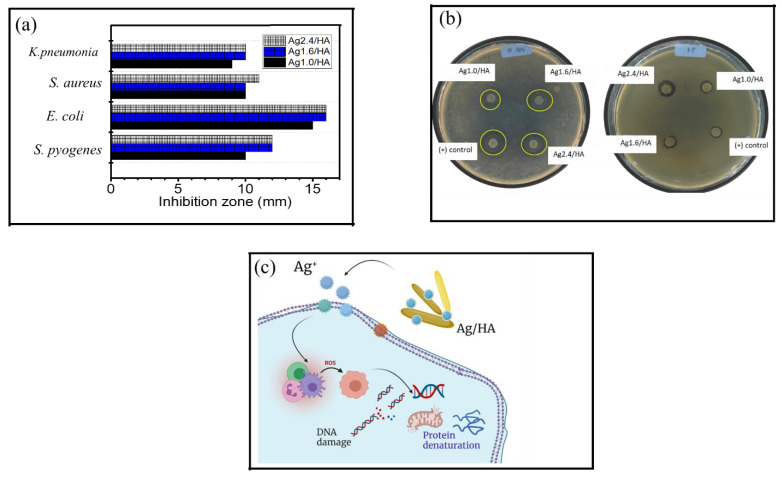
(**a**). Recapitulated inhibition zone of Ag/HA (**b**). Images from antibacterial test (**c**). Schematic representation of antibacterial activity of Ag/HA.

Antibacterial activity of the Ag/HA samples is presented by the recapitulated inhibition zone in Figure 6a, with some images from the test presented in Figure 6b. Referring to several previous works, the mechanism of antibacterial activity in Ag/HA is associated with the capability of nano-sized Ag to penetrate cell walls and create ionic conditions that prevent the formation of ATP and DNA replication of bacteria. As the ions enter the cytoplasm, there is an increase in permeability that causes bacterial death. Another mechanism is that Ag^+^ ions penetrate the cell membrane and react directly with the -SH (thiol) group of cell proteins, causing the release of ATP synthesis during cellular respiration and the loss of protons, disrupting the cell metabolism system (Figure 6c). From the inhibition zone value, varied Ag content (1.0; 1.6; and 2.4 %wt.), it is seen that the higher Ag content creates a trend in increasing antibacterial activity. Similarly, a higher activity against Gram-negative compared to Gram-positive bacteria is found [24]. In more detail, the minimum inhibitory concentration (MIC) of the samples compared to AgNPs and HA are presented in Table 1.

The data suggest that the higher Ag doped in HA expressed a lower MIC compared to HA, suggesting that the increase in nanoparticle sites interacting with the cell wall affects the bacterial membrane permeability for ongoing cell lysis [35,36]. The materials showed a stronger inhibition against Gram-negative rather than Gram-positive bacteria, as the Gram-negative bacteria have more accessible walls. With a thin peptidoglycan layer and some functional groups such as amino groups, carboxyl, and phosphate in the phospholipid structure of the cellular membrane, the wall has negative charges, which allows it to be penetrated more easily by the positive charge of AgNPs of Ag/HA composites [37,38,39]. The activity against *E. coli* from this work is higher (15 mm) compared to Ag/HA synthesized by previous researchers with 8%wt of Ag–which showed an inhibition of 10 mm [40]–and higher compared to Ag/HA synthesized using *Clitoria ternatea* extract with an inhibition zone of 12 mm [37]. However, the activity against *S. aureus* is smaller as it showed a smaller inhibition zone (10 mm) than what was recorded in the aforementioned research works of 18 mm and 12 mm, respectively. Moreover, the examination of the minimum inhibition concentration (MIC) suggests that all materials synthesized in this work have the potential to be developed as the values are comparable to previous works. For example, the MIC of all samples toward all tested bacteria are lower than what was expressed by Ag/HA prepared with Black Sumatra chicken bone [41]. In addition, the similar inhibition values for *E. coli* and *S. aureus* were achieved by higher concentrations of Ag/HA material synthesized using gallic acid as a bioreductor [42], or a higher Ag content [43]. The comparisons between the antibacterial activity with similar works are listed in Table 2. Various bioreductors such as gallic acid, *Clitoria ternatea*, and synthesis methods were reported. Compared to the Ag/HA composites obtained using gallic acid and *Clitoria ternatea* as bioreductors, the MIC values for *S. aureus* and *E. coli* from this work are smaller, indicating a higher bioactivity. However, with a similar Ag content (2%), the MIC value is lower than what was prepared by mixing and hydrothermal methods. The competitiveness of Ag/HA composite obtained in this work is related to the sustainable raw material. From these observations, it is conclusive that the Ag/HA synthesized in this work has the potential to be developed as an antibacterial material.

## 4. Conclusions

Green synthesis of silver nanoparticle-doped hydroxyapatite (Ag/HA) nanocomposite was successfully prepared. The AgNPs was synthesized by using *Curcuma longa* leaf extract as a bioreductor, and for hydroxyapatite preparation, biogenic silica derived from land snail shells was utilized. The effect of the Ag content on the physicochemical characteristics and antibacterial activity of Ag/HA was studied by employing instrumental analysis of XRD, TEM, and XPS. It was found that the green synthesized AgNPs had a particle size ranging from 2–20 nm, and was homogeneously distributed in the Ag/HA form. The effect of silver doping revealed that increasing the Ag content influenced the distribution of Ag nanoparticles. The presence of doped silver enhanced the antibacterial activity against *E. coli*, *K. pneumoniae*, *S. aureus*, and *S. pyogenes*. The results provide a reference for developing novel antibacterial materials for various applications, such as environmental applications and medical applications.

## Figures and Tables

**Table 1:** MIC of Ag/HA samples in comparison with HA and AgNPs.

Sample	MIC (μg/mL)				
	Ag1.0/HA	Ag1.6/HA	Ag2.4/HA	HA	AgNPs
*E. coli*	5	10	10	100	2
*S. aureus*	20	10	10	100	2
*K. pneumonia*	20	20	20	100	5
*S. pyogenes*	40	20	20	100	5

**Table 2:** The comparison of the antibacterial activity in similar works.

Synthesis Method of Ag/HA	Remark	Reference
Ag/HA synthesized by using gallic acid as bioreductor	The material showed an inhibition zone of around 15 mm for *E. coli*, and 9.5 mm for *S. aureus* at the concentration of 5mM	[42]
Ag/HA synthesized by 8%wt. of Ag content	The material exhibited an inhibition zone of 10 mm for *E. coli* and 18 mm for S. aureus at the concentration of 20 μg/mL	[40]
Ag/HA synthesized by using *Clitoria ternatea* as bioreductor	The material showed an inhibition zone of around 12 mm for *E. coli*, and 12 mm for *S. aureus* at the concentration of 20 μg/mL	[37]
Ag/HA synthesized by using sol-gel method	The MIC towards *E. coli*, and *S. aureus* are 15 and 25 μg/mL, respectively	[44]
Ag/HA synthesized by using black Sumatra chicken shell	The MIC towards *E. coli*, and *S. aureus* are 72 and 45 μg/mL, respectively	[41]
Ag/HA synthesized by rapid mixing method	The MIC towards *S. aureus* is 2.5 μg/mL for the Ag content of 2 % wt.	[45]
Ag/HA synthesized by using hydrothermal method	The MIC towards *S. aureus* is 7.85 μg/mL and the MIC towards *E. coli* is 3.9 μg/mL by the Ag:HA molar ratio of 3:10.	[43]

## Data Availability

Not applicable.
